# Exploring Statistical and Population Aspects of Network Complexity

**DOI:** 10.1371/journal.pone.0034523

**Published:** 2012-05-08

**Authors:** Frank Emmert-Streib, Matthias Dehmer

**Affiliations:** 1 Computational Biology and Machine Learning Lab, Center for Cancer Research and Cell Biology, School of Medicine, Dentistry and Biomedical Sciences, Queen’s University Belfast, Belfast, United Kingdom; 2 Department of Biostatistics and Computational Biology, Dana-Farber Cancer Institute, Harvard School of Public Health, Boston, Masachusetts, United States of America; 3 Institute for Bioinformatics and Translational Research, UMIT, Hall in Tyrol, Austria; Pacific Climate Impacts Consortium, Canada

## Abstract

The characterization and the definition of the complexity of objects is an important but very difficult problem that attracted much interest in many different fields. In this paper we introduce a new measure, called *network diversity score* (NDS), which allows us to quantify structural properties of networks. We demonstrate numerically that our diversity score is capable of distinguishing ordered, random and complex networks from each other and, hence, allowing us to categorize networks with respect to their structural complexity. We study 16 additional network complexity measures and find that none of these measures has similar good categorization capabilities. In contrast to many other measures suggested so far aiming for a characterization of the structural complexity of networks, our score is different for a variety of reasons. First, our score is multiplicatively composed of four individual scores, each assessing different structural properties of a network. That means our composite score reflects the structural diversity of a network. Second, our score is defined for a population of networks instead of individual networks. We will show that this removes an unwanted ambiguity, inherently present in measures that are based on single networks. In order to apply our measure practically, we provide a statistical estimator for the diversity score, which is based on a finite number of samples.

## Introduction

Complexity is a general notion that triggered a large number of studies in a variety of different fields, ranging from biology, chemistry and mathematics to physics [1]–[9]. Despite this attraction, up-to-now a generally accepted description of the complexity of an object that would allow the establishment of a quantitative measure for its characterization is still absent. Probably the best studied objects with respect to the characterization of their complexity are one- and two-dimensional strings or symbol sequences. For such objects, many approaches have been suggested to define or assess complexity quantitatively [3], [8], [10]–[18]. However, an intrinsic problem of any complexity measure is that there are alternative ways to perceive and, hence, describe complexity leading inevitably to a multitude of different complexity measures [19]. For example, Kolmogorov complexity [2], [3], [8], [20] is based on algorithmic information theory considering objects as individual symbol strings, whereas the measures *effective measure complexity* (EMC) [16], *excess entropy* [21], *predictive information* [22], *thermodynamic depth* [17] or *statistic complexity* [14] relate objects to random variables and, hence, are ensemble or population based.

In the context of networks, graph complexity measures have been suggested to investigate the complexity of chemical graphs representing molecules and chemical compounds [23]–[25]. Different types of graph complexity measures have been developed which can be broadly divided into information-theoretic and non-information-theoretic measures. Because so far it is largely unclear what structural features of a network to emphasize, hierarchical approaches for the chemical complexity consisting of several hierarchical levels of molecular complexity have been developed. One of the first attempts was due to Bertz [26] developing a hierarchical model containing both topological (i.e., branching, rings, multiple bonds) and non-topological (molecular size, symmetry, functionality, elemental composition) features; for a detailed discussion see [25]. Later, Bonchev and Polansky [27] furthered this system and described the total complexity of a chemical system by a vector approach. The components of this vector represent various features of complexity, e.g., the system size, graph topology, physical nature, metric of a system and its symmetry [27].

Also for general networks there are many network complexity measures that have been suggested [24], [28]. Many of these are based on information-theoretic principles [29]–[31]. A classical, non-information-theoretic approach is the so-called *combinatorial complexity*, introduced by Minoli [32]. This measure represents a monotonically increasing function of the factors which contribute to the complexity of a network, e.g., the number of vertices and edges, vertex degrees, multiple edges, cycles, loops, and labels [33]. Other techniques rely on determining particular substructures in graphs [24], [28]. Also Constantine et al. [34] defined the complexity of a graph to be the number of its containing spanning trees. An operator approach has been developed by Jukna [35] who defined graph complexity as the minimum number of union and intersection operations required to obtain the entire set of its edges starting from star graphs. Approaches to define the complexity of graphs based on Kolmogorov’s complexity paradigm [3] can be found in [36], [37]. Particularly, Bonchev [37] compared the Kolmogorov complexity of a graph with other measures and tackled the problem whether all these techniques can detect branching in graphs.

The major purpose of this paper is to introduce a network measure, called the *network diversity score* and to demonstrate that this measure allows to categorize networks with respect to their structural complexity. Specifically, we demonstrate that the diversity score allows to distinguish ordered, random and complex networks from each other. Further, we study 16 additional network complexity measures and find that none of these measures has similar good categorization capabilities with respect to the structural complexity of networks. In contrast to many other measures suggested so far, the network diversity score is different for a variety of reasons. First, our score is multiplicatively composed of four individual scores, each assessing different structural properties of a network. That means our overall score reflects the structural diversity of a network. Abstractly, this may be seen as the dimension of the complexity of a network. Second, our score is defined for a population of networks instead of individual networks. We will show that this removes an unwanted ambiguity, inherently present in measures that are based on single networks. To enable a practical application of the network diversity score we provide a statistical estimator for this score that is based on a finite number of networks sampled from the underlying population of networks.

This paper is organized as follows. As the definition for a structural complexity of networks suffers from similar problems as for one-dimensional symbol strings, several heuristic criteria have been proposed, with which a complexity measure should be conform [25], [27]. In order to clarify what we mean by a *complex network* we provide in section ‘Characterizing the complexity of networks’ a description of this, on which we rely in this paper. Then we describe 16 network complexity measures used for our analysis and characterize their computational complexity. In order to present the network complexity measures used in this paper, we roughly categorize them into two classes: information-theoretic and non-information-theoretic measures. Clearly, each group can be further subcategorized. For instance, we could subsume the class of pure distance-based and eigenvalue-based measures under the category of non-information-theoretic measures. As known, information-theoretic graph complexity measures [23], [38] rely on inferring a probability distribution by taking structural features of a graph into account. More precisecly, so-called partition-based and non-partition-based measures can be derived by using Shannon’s entropy, see [23], [39]. Other graph entropy measures based on using subgraph-relations can be found in [28]. Non-information-theoretic complexity measures are mostly based on transforming simple graph invariants such as vertex degrees and distance-based quantities [40] into real numbers [41], [42]. For instance, the first zagreb index [41], [42] transforms vertex degrees into a positive measure for characterizing the structure of the graph. Another class of non-information-theoretic complexity measures is based on deriving subgraphs and then transforming them into measures finally leading to a graph complexity measure, see [28]. In section ‘Network diversity score’ we define our measure and clarify conceptual differences to other approaches. In the results section we investigate all 17 network measures for a variety of different settings and compare them with each other. The paper finishes with a ‘Conclusion’ section, summarizing the obtained results.

## Methods

In this section we, first, provide a characterization for the complexity of networks as used in this paper. Then, we describe 16 network complexity measures we are using in our analysis and characterize their computational complexity. Thereafter, we introduce a new complexity measure, called *network diversity score* (NDS), and provide a motivation for its definition.

### Characterizing the Complexity of Networks

**Figure 1:**
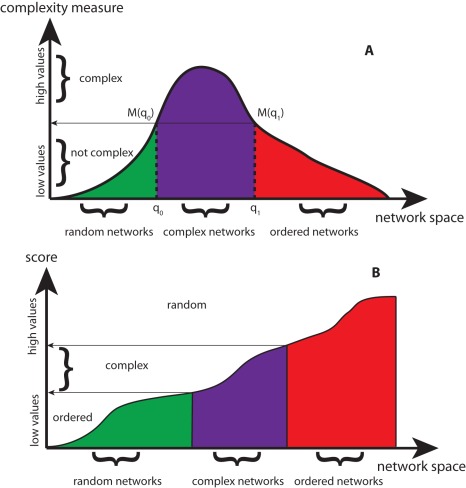
A: Visualization of the properties of a complexity measure with respect to different networks. B: Alternative complexity measure with different characteristics.

As outlined in the introduction, so far there is no universally accepted definition of complexity available that would be applicable to general objects, including networks. However, it is generally believed that a complexity measure should be capable of distinguishing complex objects from random and ordered objects. For objects generated by a physical process this complexity characterization has been given in [4], [19]. However, also for the complexity of biological systems similar assertions have been made [43]. In the following we adopt this perspective. Figure 1 A provides a visualization of this characterization, placed in the context of networks. In this figure the *x*-axis corresponds to an one-dimensional variable 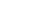 that represents networks *G* from the network space 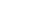, and the *y*-axis gives the value of the complexity measure 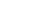. Here, the variable *q* is assumed to represent networks of a similar type smoothly. That’s why certain regions of the *x*-axis have been labeled as, ordered, complex or random. Concrete examples for such a variable is Langton’s 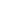 [44] for one-dimensional cellular automata or the mean connectivity *K* in random boolean networks [45].

It is important to clarify the relation between three different entities: the network *G*, the variable *q* representing a network and the complexity measure *M*. A network is an abstract object which possesses a multitude of different properties, e.g., number of nodes, degree distribution, mean path length between all nodes, to mention just a few. For this reason, a network is not easily quantifiable by a singe variable because a mapping, 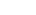, is usually not unique. For example, if we identify 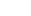 with the (global) clustering coefficient of network *G* [46], then there are many networks that have the same value of *q*. For this reason, when one maps a network *G* to *q*, the value of *q* represents actually a set of networks that map to the same value of *q*, i.e., 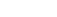 with 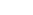. Similar arguments hold when we map a network to its complexity value, i.e., 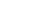. Also in this case, usually, many networks map to the same complexity value, 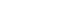 with 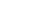. It is interesting to note that after networks have been identified as *complex*, *random* or *ordered*, by using the complexity measure *M*, the entity *q* can serve itself as a complexity measure, if it exhibits a smoothness property with respect to the underlying networks. Here, smoothness means that similar networks lead to similar values of *q*. This smoothness property allows the identification of continuous regions (intervals) of *q* values, which represent specific types of networks, as shown in Fig. 1 A.

The particular problem we want to study in this paper differs from the above. Instead of using a complexity measure *M* to categorize networks into the groups complex, random or ordered, we assume that such a categorizations for the networks is already known. From the above discussion we know that if we find a smooth measure 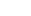, representing sets of networks that assigns to similar network types, similar values of *q*, then *q* can serve as a complexity measure. That means for networks that are labeled according to certain categories they belong to and a measure *q*, one can quantitatively assess the quality of such a measure with respect to the given labels of the networks. Hence, by using the knowledge of the labeling of different networks we can investigate the categorization abilities of a measure *q*.

In Fig. 1 B we show an alternative behavior of a complexity measure in dependence on networks. In this case, we called the values on the *y*-axis ‘score’ and not complexity measure because here a score for *complex* networks does not lead to the highest possible values but to intermediate values. However, the advantage of such a score, compared to the ones illustrated in Fig. 1 A, is that it allows to discriminate between all three network types, complex, ordered and random networks, considering the score of the networks only. Hence, there are three continuous regions of values of the score that allow to distinguish the three types of networks unambiguously. Other configurations may be possible and helpful, however, in the following, we base our analysis on this basic characterization of complexity and apply it to networks. As our numerical results will demonstrate, the principle behavior of the score sketched in Fig. 1 B is of practical relevance for our analysis (see Fig. 8 and its discussion).

### Definition of Complexity Measures

In the following we provide a brief description of the complexity measures we are using in our study. We denote by *G* a network having vertex set *V* and edge set *E*. The number of vertices is 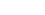 and the number of edges 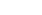. Table 1 gives an overview of the 16 complexity measures we use.

**Table 1:** Overview of the network complexity measures we use in our analysis.

Nr.	Label	Name of the measure	Reference
1.	balabanJ	Balaban *J* index	[42], [47]
2.	bertz	Bertz index	[26]
3.	bonchev2	Bonchev-Trinajstić index	[48]
4.	complexityIndex*B*	Complexity index	[24]
5.	efficiency	Efficiency complexity	[49], [50]
6.	energy	Graph energy	[51]
7.	InfoTheoGCM	Information-theoretic complexity measures	[39], [52]
8.	lapEnergy	Laplacian energy	[53]
9.	mDistDev	Mean distance deviation	[40], [42]
10.	nEdgeComplexity	Normalized edge complexity	[24]
11.	offdiagonal	Offdiagonal complexity	[54]
12.	randic	Randić connectivity index	[55]
13.	sTreeSens	Spanning tree sensitivity	[28]
14.	tInfoContent	Topological information content	[56]
15.	wiener	Wiener index	[57]
16.	zagreb	Zagreb index	[41], [42]

The label (second column) refers to a short name we use to refer to a particular measure.

#### Information-theoretic Complexity Measures

A variety of entropic measures determining their structural information content have been developed to characterize networks structurally [38]. The following measures are based on Shannon’s entropy.

- **Topological information content:**

One of the first measures was the topological information content introduced by Rashevsky [58] given by
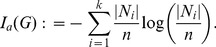
(1)


Here, 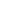 denotes the number of topologically equivalent vertices in the *i*-th vertex orbit of *G* and *k* is the number of different orbits. 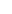 is a measure of symmetry in graphs. This measure vanishes for a fully symmetric graph such as regular graphs and attains its maximum value for asymmetric graphs. Importantly, Trucco [59] also investigated this measure and Mowshowitz [56] generalized it to determine the structural information content of graphs and studied mathematical properties thereof [56], [60], [61].

- **Bertz index:**

A more general graph complexity measure is due to Bertz and expresses the total structural information content of a graph:
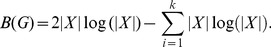
(2)


*X* is an arbitrary graph invariant such as its vertices, edges, degrees etc. 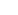 refers to its cardinality. For example, if *X* corresponds to the vertices of a network than 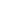 corresponds to the number of vertices. If we choose 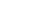, we get
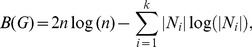
(3)


as special case.

- **Bonchev-Trinajstić index:**

By defining weighted probability schemes, one generalizes classical measures of Rashevsky and Mowshowitz [56], [58], see Eq. 1. A special measure thereof is given by
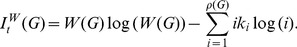
(4)


This measure is based on the Wiener-Index [57],
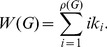
(5)


Note that the Wiener index is the sum of all distances in a graph *G*. The distances can be computed by using Dijkstra’s algorithm or any other method for calculating shortest paths in a graph [62], [63]. Here, 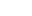 is the diameter of network *G* and 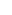 is the number of the shortest paths having length *i*.

- **Information-theoretic complexity measure based on information functionals:**

The following measure belongs to a family of graph entropy measures based on using information functionals [39]. A special measure thereof is the degree-degree association index as it is based on the special information functional 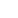, see [52]. The functional is defined by
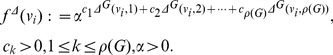
(6)


The detailed explanation and definition can be found in [52]. The degree-degree association index is defined by
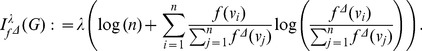
(7)



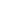
 is a scaling constant. Note that 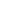 is not based on determining partitions of graph elements in a classical sense (such as 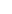) as probability values are assigned to each vertex of *G*.

- **Offdiagonal complexity:**

To define Offdiagonal complexity (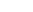) [54], let 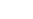 be the vertex-vertex link correlation matrix, see [54]. 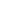 denotes the number of all neighbors possessing degree 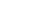 of all vertices with degree *i* [28]. 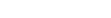 stands for the maximum degree of *G*. If one defines [28]

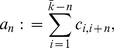
(8)


and
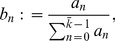
(9)



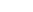
 can be defined by [28]

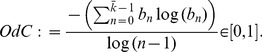
(10)

- **Spanning tree Sensitivity:**

The following measure is based on determining substructures in graphs. The spanning tree sensitivity [28] is defined by
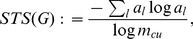
(11)with 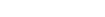, 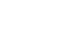, 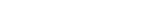 and 
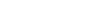
 being an ordered list of all k different 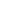. 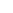 is the number of spanning trees in the graph minus the number of spanning trees of the subgraph with the edge 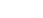 deleted. Analogously, the spanning tree sensitivity differences measure is defined as
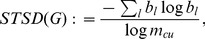
(12)with 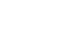, where 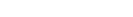 is the ordered list of all unique differences 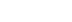.

#### Non-information-theoretic Complexity Measures

Non-information-theoretic complexity measures for networks can be defined by using arbitrary graph invariants such as distances between nodes or their degrees. In the following, we describe some important measures which have already been used in a variety of different disciplines.

- **Balaban** ***J*** **:**

The Balaban *J* index is defined as [42], [47]

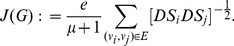
(13)



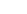
 denotes the sum of distances from vertex 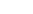 to all other vertices, i.e.,
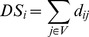
(14)whereas *D* is the distance matrix containing the shortest path lengths between all vertices measured by the Dijkstra distance [63] and 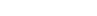 is the cyclomatic number [64].

- **Complexity index** ***B*** **:**

The complexity index *B* is a more recently developed measure due to Bonchev [24]:
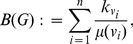
(15)


where
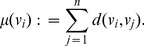
(16)


Here, 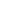 is the degree of a vertex 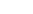.

- **Efficiency:**

Latora et al. [49], [50] developed a measure called the Efficiency complexity 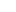 of a graph *G*. Starting from
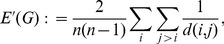
(17)


expressing the arithmetic mean of all inverse path lengths and
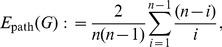
(18)


the Efficiency complexity 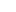 yields to
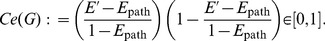
(19)

- **Mean distance deviation:**

In general, distance-based measures are straightforward to calculate with polynomial time complexity [62]. Hence, a variety of distance-based indices have been developed to characterize networks based on their topology [40], [65]. The mean distance deviation introduced by Skorobogatov and Dobrynin is defined as [40], [42]:
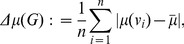
(20)


where
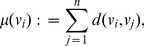
(21)


and
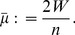
(22)

- **Normalized edge complexity:**

The normalized edge complexity using the elements of the adjacency matrix has been introduced by Bonchev [24]:
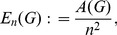
(23)


where
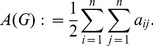
(24)


Here, 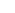 denotes the entry in the *i*-th row and *j*-th column of the corresponding adjacency matrix *A*.

- **Randić connectivity index:**

The Randić connectivity index [55]

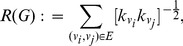
(25)has been sucessfully used as branching index. Also, *R* has been explored extensively, e.g., bounds and other extremal properties have been invesitagted in an interdisciplinary manner [66].

- **Wiener index:**

One of the first structural graph decsriptors was the Wiener-Index [57],
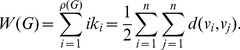
(26)



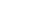
 denotes the shortest distance between 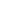 and 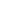.

- **Zagreb index:**

A classical degree-based index based on the vertex degree is the first Zagreb index [41], [42] defined as
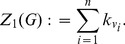
(27)



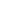
 is just the sum of the vertex degrees of *G*.

#### Eigenvalue-based Measures

By determining the eigenvalues of graph-theoretical matrices such as the adjacency matrix or the Laplacian, various measures can be obtained [51], [67].

- **Graph energy:**

Gutman [51] defined the sum of the absolute values of eigenvalues of the adjacency matrix of a graph and called the resulting quantity graph energy.
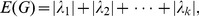
(28)where 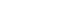 are the non-zero eigenvalues of the adjacency matrix of *G*.

- **Laplacian energy:**

Instead of using the eigenvalue of the adjacency matrix of a graph, several other graph-theoretical matrices can be used. By using the Laplace matrix, we obtain the laplacian energy [42] defined by
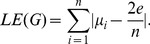
(29)


Here 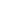 are the eigenvalues of the adjacency matrix and 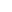 those of the Laplacian matrix of the graph.

### Computational Complexity

Calculating the complexity of networks can be computationally intense and many algorithms are even NP-complete [68]. For instance, determining the automorphism group of a general graph to compute the graph entropy measure 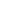 is computationally demanding as the computational complexity can be exponential [69]. In contrast, the time complexity of some information-theoretic graph complexity measures such as *B*, OdC, 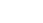 and 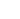 is polynomial, see [70]. Particularly the time complexity of the Bonchev-Trinajstić index 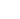 and the degree-degree association index 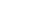 is 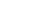 as we need to calculate all shortest paths between all vertices in the graph leading to 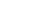. Similar statements [28], [70] for the time complexity of *J*, 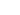 and 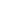 can be obtained as the complete distance matrix needs to be calculated. Simple topological network measures, such as the Wiener and Randić index also possess polynomial time complexity as their calculation rely on matrix computations based on graph invariants.

The time complexity of determining the zeros (eigenvalues) [71] of graph polynomials [51] such as the characteristic or distance polynomial is polynomial too. For instance, by using the adjacency matrix to calculate the characteristic polynomial of a graph, we obtain its eigenvalues 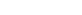 in polynomial time. From this, measures such as the graph energy *E* and the laplacian energy *LE* can be calculated efficiently.

### Network Diversity Score

In the following we define a network measure we call the *network diversity score* (NDS). Our score is based on 4 variables:
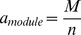
(30)

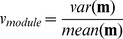
(31)

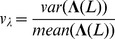
(32)

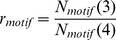
(33)


Here, *M* is the number of modules in a network *G* and *n* is the number of vertices of this network. The vector 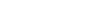 contains the size of the modules, i.e., 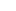 gives the size of the i-th module, which corresponds to the number of nodes in this module. To identify the modules in a network we use a method called *Walktrap* [72] which finds modules based on random walks similar to [73], [74]. An advantage of this method over many others is its efficient computational complexity, given by 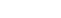 (e: number of edges, n: number of vertices). The vector 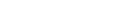 in Eqn. 32 represents the eigenvalues of the Laplace matrix *L* of network *G* [75], whose components are defined by
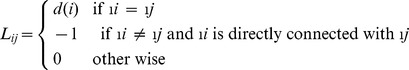
(34)


Here, 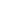 is the degree of node *i* in *G*. Finally, 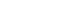 and 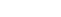 correspond to the number of motifs of size 3 and 4 found in network *G* [76]. That means 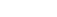 is the number of different motifs one can find in *G* having *i* nodes.

Based on the above four variables, we define the *individual diversity score* for a network *G* by
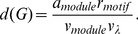
(35)


We call this measure *individual diversity score* because it can be calculated for a single network *G*. The individual diversity score 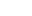 assesses one network *G* and assumes values in 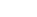. Based on 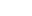 we define the *network diversity score* (NDS), 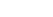, for a population of networks 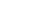 by
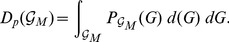
(36)


Here, 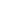 denotes the population of networks that belong to the same network model and 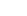 is a probability density over this population. For example, this could correspond to the random network model generated with the Erdös-Réyni model [77], [78]. Or it could be the set of all scale-free networks generated with the preferential attachment algorithm [79], [80]. Or the population could contain all networks that have the same degree, e.g., a lattice with periodic boundary conditions. That means the population of networks 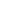 can be either defined by a stochastic process that generates the networks in the population or by structural properties of the networks themselves.

In order to obtain an approximation of the measure 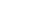, which can be applied to a finite set of networks, we define the *network diversity score* for a sample of size 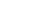 from the population 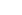 by the estimator,
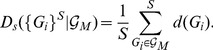
(37)


Assuming that the *S* networks are independently sampled from the population 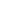 than, according to the central limit theorem [81],
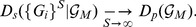
(38)


For our numerical investigations we use the estimator given in Eqn. 37.

The diversity score represents the idea that a network is a high-dimensional object. Specifically, we consider the 4 variables 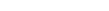 and 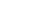 as important. The variable 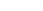 provides information about the module density of a network. For complex networks we would expect to find more modules than for random networks because modules are an expression of a general organizational principle of a network. The variable 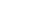 is a rate about the growth of motifs within a network. From numerical results we observed that ordered networks have the highest, complex network have intermediate and random networks have the lowest values of 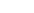. The variable 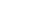 is similar to a CV (coefficient of variation) value which measures the variability of network sizes with respect to the mean size of a module. Random networks are expected to have a low variability of module sizes but also a low mean module size whereas complex networks should have a higher variability of module sizes but also a higher mean module size. The variable 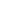 is similar to 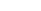 but for the eigenvalues of the Laplace matrix *L*. We studied many combinations of these 4 and other variables and found from numerical investigations that the individual density score in Eqn. 35 results in the best separation of random, complex and ordered networks.

#### Motivation for the network diversity score

The underlying rational of our measure is based on the following observations. First, studies investigating the complexity of various types of objects, e.g., one-dimensional strings, led to the introduction of a large number of different complexity measures. However, up-to-now there is no general agreement that the *right* measure is among the introduced ones. For networks, we are facing a similar situation that may be potentially even more severe. For this reason, we are proposing a composite measure that is not just based on the evaluation of *one* structural principle, but on the *combination* of several ones. Hence, their combinatorial usage abates the need for each individual measure to represent the *right* complexity measure. In the results section, we will numerically demonstrate that such a composite measure leads in fact to very good results.

A second reason that motivated us to introduce our measure is best described by the following illustration. Suppose, one defines networks as ‘random’ when they have been generated with the random network model, suggested by Erdös-Réyni and Gilbert [77], [78], and as ‘complex’ when they have been generated with the preferential attachment algorithm [79], [80]. Then, there exists a non-vanishing probability to generate a random network with the random network model that is also complex. However, this is counter intuitive. Let us consider an example for this problem. Suppose, a network 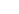 has been generated with the random network model and a second network 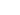 has been generated with the preferential attachment algorithm. Then, with a certain probability, 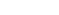 (with the meaning 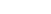) holds, because the random network model can, in principle, generate all possible network structures. More precisely, if the undirected network 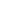 contains *e* edges (denoted by 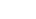) and *n* vertices then it contains 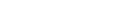 missing edges (non-edges). That means the probability, *w*, for the random network model to generate a particular network with *e* edges is given by
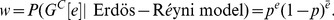
(39)


**Figure 2:**
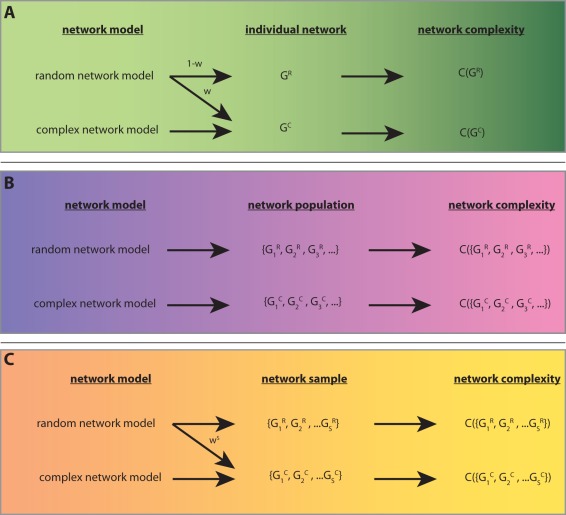
Connection between network model, networks and a complexity measure assessing either the complexity of individual networks (A), a population of networks (B) or a sample of networks (C).

Here, 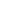 is the probability to have *e* edges in 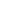 and 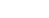 is the probability to have 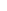 non-edges in 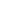. That means, assigning a complexity value to individual networks leads to a loss of the unique connection between the complexity of the network and the underlying network model that generated this network. This is visualized in Fig. 2 A. In this figure, *w* corresponds to the probability that the random network model generates a complex network 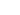. Starting from the complexity value of a network, right hand side of the figure, one sees that it is possible to conclude that 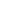 has been either generated with a random network model or with a complex network model. For reasons of simplicity, we used in the above explanation only two network models, however, an extension to more models is straight forward, but makes the explanations more laborious. It should be clear that in such an extended scenario, the potential for an ambiguity between the complexity of individual networks and the network generating models is even amplified.

In order to avoid this problem, we base our network score on the principle visualized in Fig. 2 B. Due to the fact that the complexity is assessed for a *network population*, generated by a network model, there is no confusion with respect to the underlying network model that generated the population, because the complexity measure can rely on the information provided by the whole population and not only by an instance thereof. Practically, we approximate such a population measure by using a finite sample of networks, as shown in Fig. 2 C. For a finite sample consisting of *S* networks, there is also a non-vanishing probability to result in an ambiguous connection between the complexity 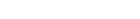 and the underlying network model that generated the network sample, visualized in Fig. 2 C. However, this probability is only 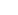, compared to *w* for a complexity measure relying on a single network. In the limit for 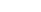 this probability goes to zero and model C becomes model B for any 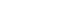. Hence, using a sample of size *S* reduces the potential for an ambiguity leading to a miscategorization by a factor of 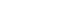. For example, if 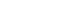 and the sample size is only 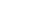 than this factor is already 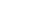.

We would like to emphasize that the above explanations are intended as a motivation of our approach and not as a numerical analysis of the most general situation conceivable. In this respect, the probability *w* given in Eqn. 39 needs to be adapted for more general situations. However, regardless of its precise value, *w* will be always larger than zero and the principle discussion above translates seamlessly to more involved conditions. In the next section, we provide a numerical analysis for a large variety of different networks.

## Results

**Figure 3:**
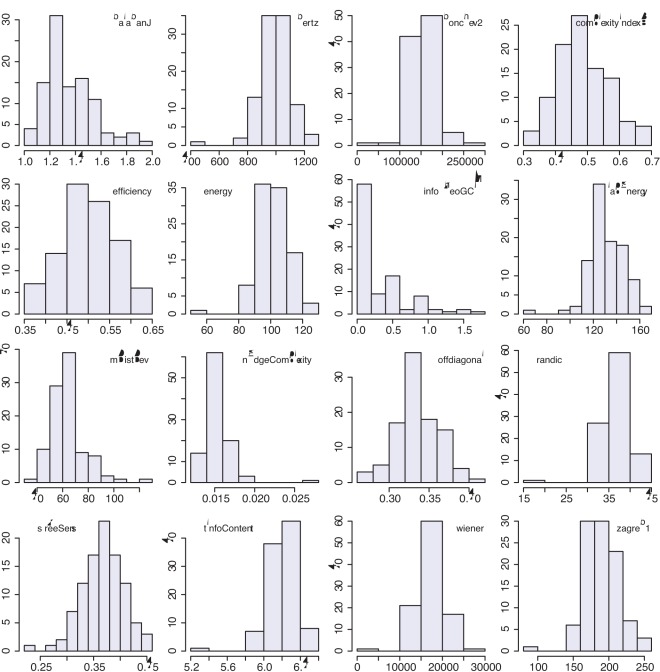
Evaluation of 100 random networks generated with the random network model, 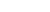 and 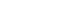. Each histogram shows the results for one network measure; see the legend for the name of the measure.

We begin our analysis by investigating the statistical variability of the 16 network complexity measures listed in Tab. 1. In Fig. 3 we show results for 100 networks generated with the random network model [77], [78] for the parameters 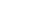 and 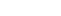. Here *n* corresponds to the number of nodes in a network and the parameter 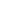 is the probability with which two nodes are connected by an edge. Each histogram shows the result for one complexity measure, as indicated by the name in the legend. The *x*-axis corresponds to the value of the respective complexity measure and the *y*-axis gives the frequency of observed values. It is important to note that despite the fact that all random networks have been generated for the same network parameters, *n* and 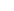, the resulting complexity measures do not provide identical results but fluctuate. We repeated this analysis for different parameters of the random network model and also for different network types, i.e., for complex networks. For all studied cases, we found qualitatively similar results. This reveals a common conceptual drawback of all these network measures because none of the measures is considered as a random variable. However, due to the fact that a network is sampled from an underlying population, this network varies structurally, and, hence, also the network measure, as seen in Fig. 3. That means ignoring this fact is counter productive and results in a loss of interpretability of these network measures, as will be demonstrated later in this section (see Fig. 7). As explained in section ‘Network diversity score’, a random network model is in principle capable of generating all possible types of networks, including ordered and complex networks, however, only with a certain probability. Due to the fact that all measures assess only one network, which has been randomly sampled from the underlying population of a network model, the sampled network conveys the variability of network structures of the population to the network measure itself.

**Figure 4:**
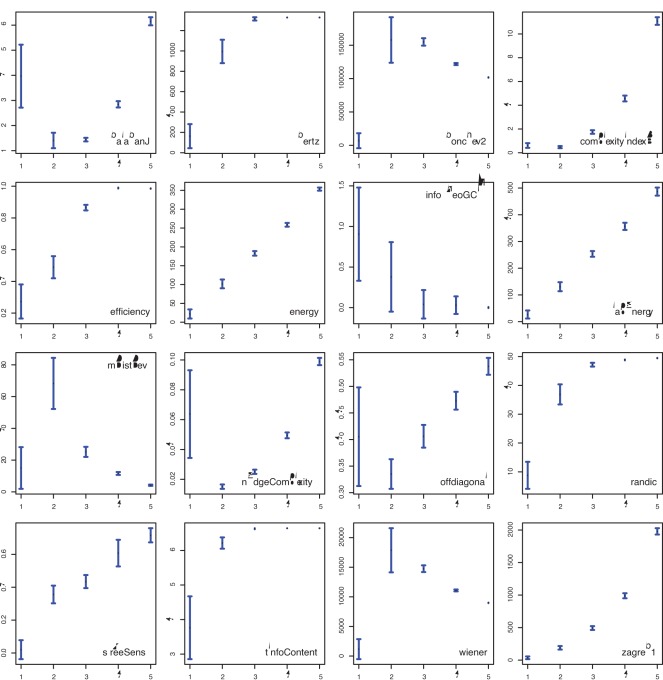
Random network model: Dependence of the complexity measures (*y*-axis) on 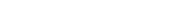 (*x*-axis).

**Figure 5:**
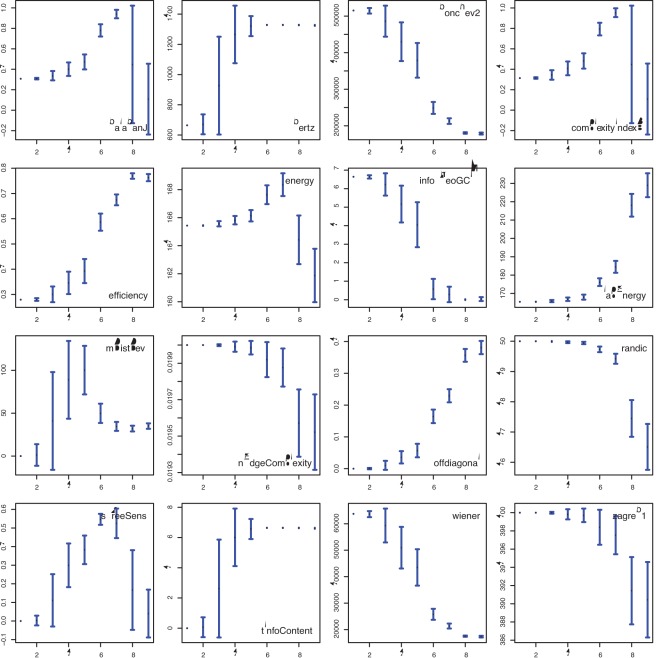
Small-world network model: Dependence of the complexity measures (*y*-axis) on 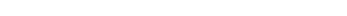 (*x*-axis).

In the Figs. 4 to 5 we show results for two different network models and the influence of model parameters on the 16 complexity measures. In Fig. 4 we show results for a random network model with a connection probability between nodes of 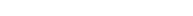 (*x*-axis). Fig. 5 shows results for a small-world network model [82] for a rewiring probability of 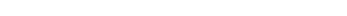 (*x*-axis). In these figures, the mean value and the standard deviation of a complexity measure (y-axis) is shown in dependence of the model parameter (*x*-axis).

Fig. 4 demonstrates that among the 16 complexity measures, one can observe four qualitatively different types of behavior. The four observed behavior are: (1) a monotonous increase in the complexity value (complexityIndexB, efficiency, energy, lapEnergy, randic, sTreeSens, tInfoContent, zagreb1), (2) a monotonous decrease in the complexity value (infoTheoGCM), (3) increasing complexity values followed by decreasing values (bonchev2, mDistDev, wiener), (4) decreasing complexity values followed by increasing values (balabanJ, nEdgeComplexity, offdiagonal). This indicates that different network measures have entirely different characteristics due to different structural features of the network they capture. Further, we observe that all measures, except infoTheoGCM, result in non-overlapping values for different model parameters which means that different values of 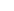 lead to significantly different values of the corresponding complexity values. This is important to note since all networks generated with the random network model for different values of 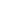 are random networks.

The results for the small-world network model, shown in Fig. 5, are principally different to the results shown in Fig. 4, because for different values of 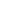 we obtain different network types. Specifically, we obtain ordered (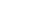), complex (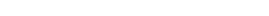) and random networks (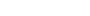). This is different to the results for the random network model because different model parameters result always in a random network, whereas for a small-world network model, different model parameters lead to a different type of a network. Among the 16 network measures, 5 demonstrate a discriminative behavior with respect to the three different network types (balabanJ, complexityIndexB, energy, mDistDev and sTreeSens). That means these 5 measures exhibit for complex networks (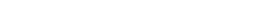) noticeably different values than for ordered and random networks.

**Figure 6:**
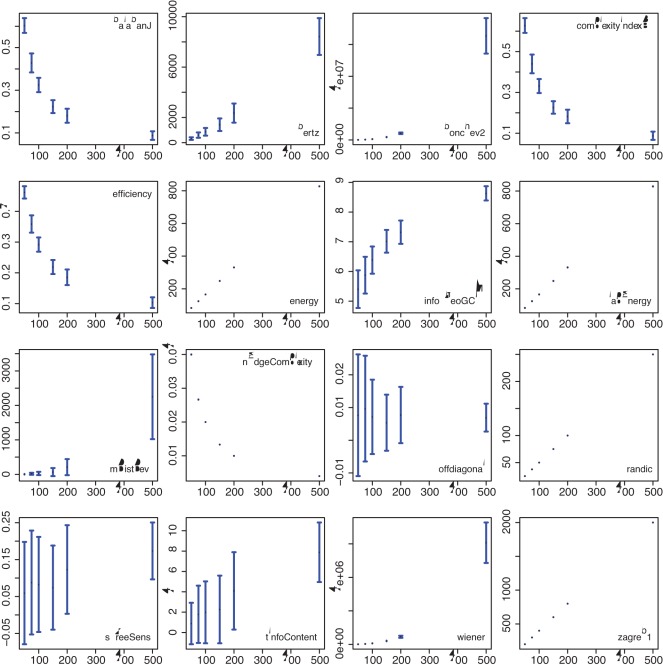
Dependence of the complexity measures on the size *n* (*x*-axis) of small-world networks.

In Fig. 6 we show results about the influence of the network size *n*, ranging from 100 to 500 nodes, on the complexity measures. Because the type of a network does not change for a different size of the network, one would *ideally* expect constant values of the network measures for all different network sizes. The only measures that are approximately constant are offdiagonal and sTreeSens because their mean complexity values do not change much if taking the standard deviation of the measure into consideration. All other measures are significantly effected by the size of the networks. This hints that the size of a network is an important parameter. To simplify the following analysis, we study only networks of a fixed size.

So far, we studied only individual network models for a variety of different parameters these models depend on. Now, we investigate a mixture of different network models. More specifically, we generate a set, 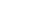, consisting of 1500 networks, each with 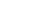 vertices. This set is composed of 200 ordered networks, 600 random networks and 700 complex networks. The set of complex networks is itself a mixture of scale-free networks, with different parameters of the power of the preferential attachment model 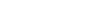, and small-world networks, with a rewiring probability of 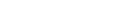. For the set of random networks we used different parameters to connect vertices with an edge, namely, 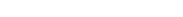. Also, we generated random networks with the small-world model by setting the rewiring probability to 1.0. That means the resulting set of networks 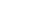 is heterogeneous with respect to the generation of the used networks. The median number of edges of these sets of the ordered, random and complex networks is 200 for each network type and their standard deviation is 109,43 and 60. The same data set will later be used to study the network diversity score (see Fig. 8).

**Figure 7:**
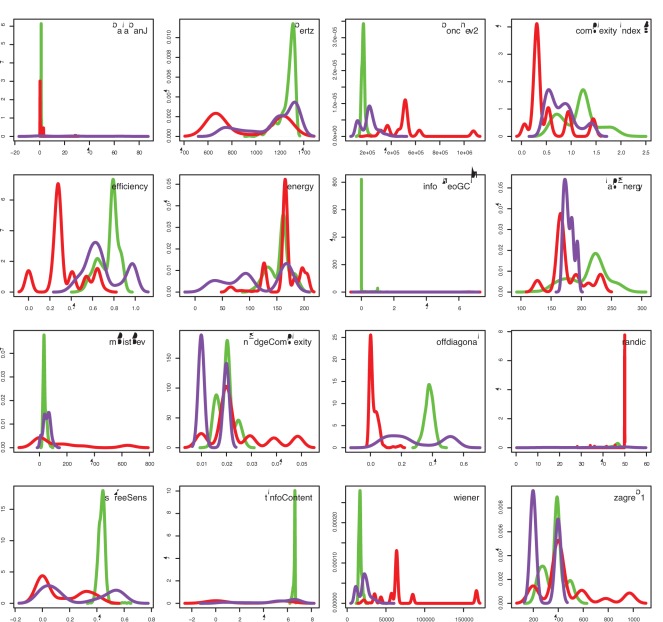
Density of the 16 complexity measures for ordered (red), complex (purple) and random (green) networks.

Application of the 16 complexity measures to 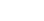 leads to the results shown in Fig. 7. These figures show the probability density of the complexity values (*y*-axis) in dependence on the complexity values of the networks (*x*-axis). The three different colors correspond to ordered (red), complex (purple) and random (green) networks. The ideal behavior of a complexity measure we would like to observe is a separation of the three different network types, which means the density of the complexity values for ordered, complex and random networks should only marginally be overlapping to enable a meaningful categorization of the three network types. Considering the obtained numerical results in Fig. 7 from this perspective we find that only the offdiagonal complexity allows, at least to a certain degree, to separate the three network types from each other. The densities of all other measures do not separate at all. The problem with the density for the offdiagonal complexity is not only that it is bimodal for complex networks but also that there is still a considerable overlapping of complex (purple) and random networks (red).

**Figure 8:**
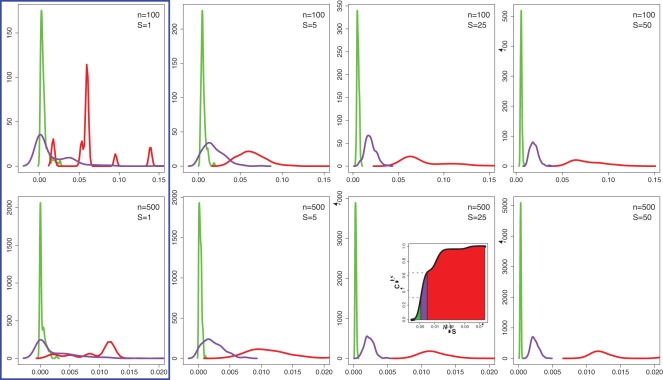
Density of the diversity score for ordered (red), complex (purple) and random (green) networks. The first row shows results for networks with 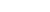 nodes and the second row for 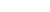 nodes. The four columns correspond to the four sample sizes 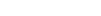.

Next, we investigate the behavior of the network diversity score, 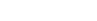, given in Eqn. 37. In the top row in Fig. 8 we show the results for the application of the diversity score to 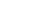. Due to the fact that our complexity score depends on the sample size *S*, the four columns in Fig. 8 correspond to four different sample sizes (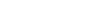). Hence, the number of different networks used for these four cases are 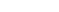 which equals to 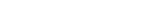 networks. We would like to emphasize that for 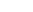, the estimator 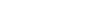 gives the worst possible approximation for the *density score* 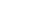. This case is not included to suggest it is a potential choice of *S*, instead, it is included to demonstrate the strength of a population effect for values of 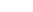. For this reason, we highlight the difference of the case 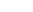 from the others, by framing the first column in Fig. 8 by a blue rectangle to indicate that it is not meant as a suggested value for the sample size.

From Fig. 8 one can see that for increasing values of the sample size *S*, the three network types - ordered networks (red), complex networks (purple) and random networks (green), respectively their densities become more and more separated from each other, as desired. But even for the sample size 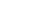, the results for the diversity score are improved compared to the offdiagonal complexity, which was the best performing measure of all 16 network measures. The second row in Fig. 8 shows a similar analysis, however, for networks having 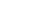 nodes for which we generated another set of networks 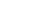 containing 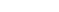 networks. For 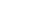 we observe an even clearer distinction of the three network types, which separate for 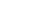 perfectly from each other. We would like to emphasize that due to the nature of the network diversity score, which is population based, a comparison with any of the 16 network measures is uneven because none of these measures can be influenced by the sample size *S*. On the other hand, a sample of networks of size *S* contains valuable information that can be exploited to increase the discriminative abilities of a measure, as demonstrated in Fig. 8. This provides evidence that the conceptual idea of a population based measure, proposed in this paper, enhances the performance of a measure to separate networks from different categories.

On a note of caution, we would like to emphasize that the discriminating ability of the diversity score is *not solely* due to its population character, instead, it is due to the combination of its population character and the *individual diversity score*, 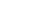, (see Eqn. 35), on which 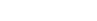 is based. From Fig. 8 one can learn about the influence of the sample size, but it does not give information about the influence of the *individual diversity score*. For this reason, we investigated the influence of the *individual diversity score* by altering its definition. For example, using only a subset of the four variables on which 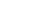 is based on (see Eqn. 30 to 33), we found that a population based version of such a measure does actually not lead to the discrimination of different network types. Hence, only the combination of an appropriate *individual diversity score* with a population approach results in the favorable characteristics of the diversity score.

In the section ‘Characterizing the complexity of networks’ we provided a characterization of complexity. The connection between this characterization, as given in Fig. 1, and our results in Fig. 8, is given by the cumulative distribution function (CDF) [81] of the densities in Fig. 8. Exemplarily, we show the CDF for 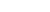 and 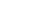. Hence, the score (*y*-axis) in Fig. 1 can be identified with the cumulative distribution function of the probability density of the diversity score.

**Figure 9:**
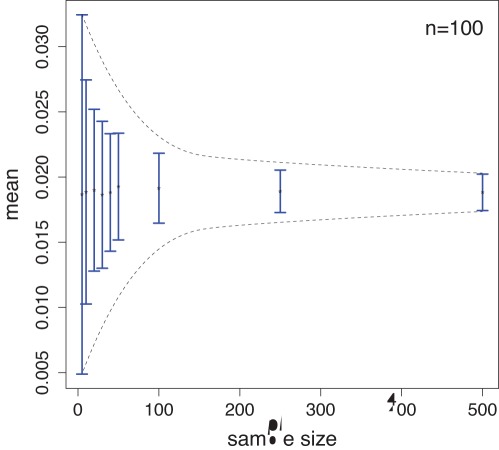
Influence of the sample size *S* on 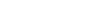 for networks of size 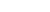.

Finally, we show in Fig. 9 the influence of the sample size *S* on the mean individual diversity score 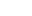, corresponding to 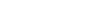, for networks of size 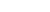. These results show that this mean value is largely constant for different values of the sample size *S* demonstrating that the unbiased estimator [83] given by Eqn. 37 provides good estimates in practice, even for small sample sizes. In addition, this figure demonstrates that very small sample sizes are not recommendable to use because the expected variability of the estimates is quite large.

### Application to Real Networks

Finally, we apply the network diversity score to four real networks. We use two social networks representing coauthorship networks between scientists working in high-energy physics (hep, 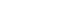) [84] and network science (net, 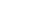) [85], a technological network representing the Western States Power Grid of the United States (power, 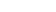) [82] and a biological network representing the protein-protein interactions in *Helicobacter pylori* (hpylo, 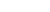) [86], which is a bacterium that can be found in the stomach. The number in brackets refers to the number of nodes in the giant connected component of these networks, we use in the following for our analysis.

Because we have only one network for each of these four networks to which we can apply the network diversity score, we utilize the following property of complexity. It is generally assumed that one aspect of the complexity of an object is the presence of a hierarchical organization structure [10], [87], [88]. This implies that not only the whole object itself is complex but also a sufficiently large components of it. For our analysis, we utilize this by randomly selecting subnetworks from a network *G*. That means, we obtain a sample of *S* networks from one network by generating randomly subnetworks with *n* vertices from *G*. This way we obtain a sample of networks 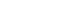, whereas each network 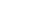 has been sampled from the network *G*, i.e.,
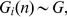
(40)


that approximates a sample from an underlying network model. Practically, we generate the subnetworks by a random walk. Starting from an initial vertex that is randomly chosen from all vertices of the network 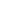, a subnetwork is defined by the first 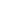 unique vertices visited by the random walk. This allows, first, to generate a sample of networks from a network model although only one network is available. Second, the size of each network can be set to a fixed value *n*. This allows the comparison of networks with a different size, because the size of the networks in the samples 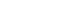 have all the same number of vertices.

**Figure 10:**
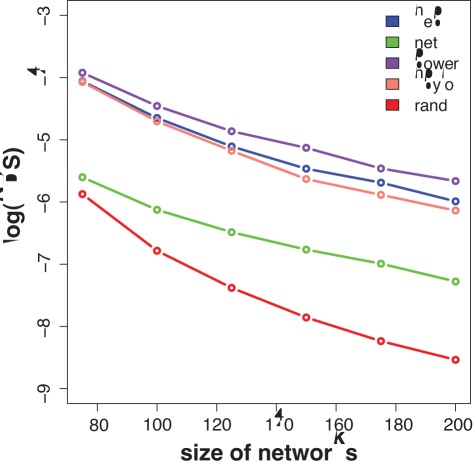
Logarithm of the network diversity score in dependence on the size of the sampled subnetworks. Every point on these curves is averaged over 100 independent samples of size *S* = 10.

In Fig. 10 we show the results for these four networks. In addition, we included results for random networks (red curve) generated with the Erdös-Réyni model. The *x*-axis gives the size of the subnetworks, *n*. The sample size for this analysis was 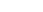 and we averaged all results over 100 independent samples. That means for Fig. 10 we analyzed a total of 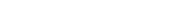 networks. Overall, one can see that random networks lead to the lowest values of the density score and for subnetworks of size 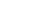 the distances between the individual networks are largely constant. This indicates that for the studied networks subnetworks of size 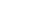 are sufficiently large to capture the complexity of the whole networks.

## Discussion

In this paper we investigated the behavior of 17 network measures with respect to their ability to categorize the structural complexity of networks systematically. Our analysis demonstrates that constructing a network measure in a way that it averages over a sample of networks from a population, enhances its capabilities to categorize different types of networks significantly. From our numerical results follow that this averaging property of the diversity score is key in order to achieve a perfect separation of the three different network types, ordered, complex and random networks, we investigated in our analysis. The crucial point here is that this averaging property reduces the importance of finding the *right* network measure that quantifies exactly what is meant by the structural complexity of a network. Due to the fact that the *right* network complexity measure is not known, we defined the diversity score multiplicatively composed of four individual scores, each one assessing different structural properties of a network. Hence, the combination of a network diversity score, which does not focus on a single structural property of a network but on multiple ones, together with the averaging over a sample of networks from a population, leads to a network measure that appears to be well adopted to the proposed task. We would like to emphasize that there are other complexity measures that also include the underlying population in the definition of the measure [14], [16], [17], [21], [22], however, all of these complexity measures have only been studied in the context of symbol sequences.

On a theoretical note, the averaging over a sample of networks from a population does not only have a very beneficial influence on the numerical categorization of different types of networks, but removes also a conceptual ambiguity present in all measures that assess only individual networks with respect to their complexity. As discussed in the ‘Methods’ section, a random network model is capable of generating complex networks too. Hence, theoretically, it is possible to generate different types of networks with the random network model. This leads inevitably to a miscategorizations of networks. In contrast, the diversity score proposed in this paper reduces this ambiguity by a factor of 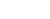, with *S* being the sample size.

The categorization of networks with respect to their structural complexity is not only interesting for theoretical, but also practical reasons. For example, in molecular biology it is generally assumed that molecular interactions between proteins and molecules generate the biological function of cells and give raise to the phenotypic appearance of organisms. Due to the fact that a graphical representation of such molecular interactions is given by gene networks, it has been suggested to compare these networks structurally in order to identify aberrations of molecular functions [89]–[91]. As an extension of the above approach it seems natural assessing the structural complexity of gene networks, e.g., of regulatory networks, to distinguish different stages of complex diseases, like cancer or cardiovascular disease, from each other. For example, gene expression data from DNA microarrays could be used to infer a regulatory network for each patient which belongs to a certain stage or a grade of a disease. Then such a disease grade can be considered as a category from which the patients and their respective networks are sampled. In this way, our network score can be applied to compare patients from different disease stages or grades with each other. Given the pace with which the data in molecular biology increase due to steady technological innovations, one can expect such data sets to be available within the near future. Other, potential areas of application are the categorization of financial networks [92]–[94] or neural networks [95], [96].
